# Reference charts for first‐trimester placental volume derived using OxNNet

**DOI:** 10.1002/uog.29300

**Published:** 2025-08-01

**Authors:** S. Mathewlynn, L. Nicolatino Starck, Y. Yin, M. Soltaninejad, M. Swinburne, K. H. Nicolaides, A. Syngelaki, A. Galán Contreras, S. Bigiotti, E.‐M. Woess, S. Gerry, S. Collins

**Affiliations:** ^1^ Nuffield Department of Women's and Reproductive Health University of Oxford Oxford UK; ^2^ Oxford University Hospitals NHS Foundation Trust Oxford UK; ^3^ Fetal Medicine Research Institute, King's College Hospital London UK; ^4^ MRC Integrative Epidemiology Unit (IEU) University of Bristol Bristol UK; ^5^ Population Health Sciences, Bristol Medical School University of Bristol Bristol UK; ^6^ Centre for Statistics in Medicine University of Oxford Oxford UK; ^7^ Birmingham Women and Children's NHS Foundation Trust Birmingham UK

**Keywords:** artificial neural network, crown–rump length, first‐trimester, gestational age, placenta, reference values, three‐dimensional ultrasonography, volume measurement

## Abstract

**Objective:**

To establish a comprehensive reference range for OxNNet‐derived first‐trimester placental volume (FTPV), based on values observed in healthy pregnancies.

**Methods:**

Data were obtained from the First Trimester Placental Ultrasound Study, an observational cohort study in which three‐dimensional placental ultrasound imaging was performed between 11 + 2 and 14 + 1 weeks' gestation, alongside otherwise routine care. A subgroup of singleton pregnancies resulting in term live birth, without neonatal unit admission or major chromosomal or structural abnormality, were included. Exclusion criteria were fetal growth restriction, maternal diabetes mellitus, hypertensive disorders of pregnancy or other maternal medical conditions (e.g. chronic hypertension, antiphospholipid syndrome, systemic lupus erythematosus). Placental images were processed using the OxNNet toolkit, a software solution based on a fully convolutional neural network, for automated placental segmentation and volume calculation. Quantile regression and the lambda‐mu‐sigma (LMS) method were applied to model the distribution of FTPV, using both crown–rump length (CRL) and gestational age as predictors. Model fit was assessed using the Akaike information criterion (AIC), and centile curves were constructed for visual inspection.

**Results:**

The cohort comprised 2547 cases. The distribution of FTPV across gestational ages was positively skewed, with variation in the distribution at different gestational timepoints. In model comparisons, the LMS method yielded lower AIC values compared with quantile regression models. For predicting FTPV from CRL, the LMS model with the Sinh–Arcsinh distribution achieved the best performance, with the lowest AIC value. For gestational‐age‐based prediction, the LMS model with the Box–Cox Cole and Green original distribution achieved the lowest AIC value. The LMS models were selected to construct centile charts for FTPV based on both CRL and gestational age. Evaluation of the centile charts revealed strong agreement between predicted and observed centiles, with minimal deviations. Both models demonstrated excellent calibration, and the *Z*‐scores derived using each of the models confirmed normal distribution.

**Conclusions:**

This study established reference ranges for FTPV based on both CRL and gestational age in healthy pregnancies. The LMS method provided the best model fit, demonstrating excellent calibration and minimal deviations between predicted and observed centiles. These findings should facilitate the exploration of FTPV as a potential biomarker for adverse pregnancy outcome and provide a foundation for future research into its clinical applications. © 2025 The Author(s). *Ultrasound in Obstetrics & Gynecology* published by John Wiley & Sons Ltd on behalf of International Society of Ultrasound in Obstetrics and Gynecology.

## INTRODUCTION

Ultrasound‐derived first‐trimester placental volume (FTPV) has emerged as a promising biomarker for several significant pregnancy‐related conditions. The positive correlation between placental size at delivery and birth weight is well established, and growing evidence suggests a relationship between low FTPV and subsequent markers of fetal growth restriction (FGR)^1^, ^2^, ^3^, ^4^, ^5^. Additionally, several studies have demonstrated an association between low FTPV and the development of pre‐eclampsia^1^, ^2^, ^6^, ^7^, ^8^, and there may also be observable differences in FTPV in pregnancies in which the mother later develops gestational diabetes^9^.

To calculate FTPV, the placenta must first be segmented from a three‐dimensional (3D) ultrasound image. Manual and semi‐automated segmentation methods are operator‐dependent and time‐consuming, posing significant barriers to large‐scale research and clinical application^10^. However, advances in artificial intelligence offer a promising solution in the OxNNet toolkit, a software solution based on a fully convolutional neural network, developed at the University of Oxford, Oxford, UK, which enables the complete automation of placental volume calculation. The development, structure and performance of the OxNNet toolkit have been described previously^11^, ^12^. This innovative software solution makes it feasible to perform placental segmentation and volume calculation on a large scale.

Currently, there is limited understanding of what constitutes a normal placental volume during the first trimester, which presents a significant barrier to fully comprehending its clinical significance. Our objective was to establish a comprehensive reference range for the OxNNet‐derived FTPV, based on values observed in healthy pregnancies. This reference range should serve as a foundation for future research and facilitate the exploration of FTPV as a potential screening biomarker in prenatal care.

## METHODS

### Study design and setting

This study used data collected for the First Trimester Placental Ultrasound (FirstPLUS) study, a longitudinal observational cohort study of unselected women who underwent first‐trimester screening at the Harris Birthright Research Centre for Fetal Medicine, King's College Hospital NHS Foundation Trust, London, UK, between March and November 2022. Participants were approached at the time of their routine ultrasound assessment between 11 + 2 and 14 + 1 weeks' gestation. Alongside routine care, 3D ultrasound imaging of the placenta was performed. Following the single study visit, routine maternity care was provided. Subsequent pregnancy outcomes were extracted from the electronic patient record. The study was conducted in accordance with Strengthening the Reporting of Observational Studies in Epidemiology (STROBE) guidelines^13^.

Ethical approval for the FirstPLUS study was granted by the West Midlands – Solihull Research Ethics Committee on 8 March 2022 (reference: 22/WM/0039). All participants provided informed written consent to take part in the study and were free to cease participation at any stage.

### Population

Women attending for the first‐trimester combined test were approached for participation. Those willing and able to give informed consent to participate were included, provided the following criteria were met: singleton pregnancy; age ≥ 18 years; presenting for the first‐trimester combined screening test between 11 + 2 and 14 + 1 weeks' gestation; able to understand written or verbal English, or able to access appropriate methods of translation; and not thought to be at risk, under stress or limited in their ability to participate in the study activities in the opinion of the investigators. Cases were excluded if any of the following criteria were met: multiple pregnancy (more than one viable fetus) identified at the time of the scan; non‐viable pregnancy or uncertain viability (no detectable heartbeat); pregnancy with major defect identified at the time of the scan; pregnancy found subsequently to be chromosomally abnormal as a result of prenatal or postnatal testing; and pregnancy for which the combined test could not be completed (e.g. it was not possible to obtain a nuchal translucency measurement).

For construction of the reference charts, a subgroup of the FirstPLUS cohort was analyzed. The objective was to analyze a group of pregnancies in which the placental volume was unlikely to have been influenced by pathology, thereby producing a proscriptive reference (a reference that reflects the range of values observed in a healthy population) as opposed to a descriptive reference (a reference that reflects the entire population, including those with pathology). In other words, we aimed to establish the normal range of FTPV in apparently healthy pregnancies. Therefore, we excluded from this analysis cases in which any of the following criteria were met: miscarriage, stillbirth, termination of pregnancy, neonatal death, preterm birth, major fetal anomaly identified subsequent to the research scan, evidence of FGR, neonatal unit admission, maternal diabetes mellitus (pre‐existing or gestational), maternal hypertensive disease (chronic hypertension, gestational hypertension or pre‐eclampsia), antiphospholipid syndrome or systemic lupus erythematosus. Cases with incomplete outcome data were also excluded. Cases were not excluded based on small‐for‐gestational age or large‐for‐gestational age birth weight alone.

### Definitions

FGR was defined as birth weight < 3^rd^ centile, or birth weight < 10^th^ centile with confirmed Doppler abnormality at the time of the final ultrasound scan. Doppler abnormality was defined as umbilical artery pulsatility index > 95^th^ centile or cerebroplacental ratio < 5^th^ centile at the final ultrasound assessment before birth, according to the reference ranges of Ciobanu *et al*.^14^. Birth‐weight centiles were calculated using the Fetal Medicine Foundation (FMF) population birth‐weight charts^15^. Pre‐eclampsia and gestational hypertension were defined according to the guideline of the American College of Obstetricians and Gynecologists^16^. Gestational diabetes mellitus was diagnosed using the oral glucose tolerance test, according to the timing and diagnostic thresholds recommended by the National Institute for Health and Care Excellence^17^. Preterm birth was defined as birth at < 37 + 0 weeks' gestation. Stillbirth was defined as birth with no signs of life at ≥ 24 + 0 weeks' gestation. Miscarriage was defined as ultrasound‐confirmed *in‐utero* fetal demise, or birth with no signs of life, at ≤ 23 + 6 weeks' gestation.

### Assessment of gestational age

Pregnancies were dated according to their crown–rump length (CRL) measurement^18^, except in cases of assisted reproduction, for which the date of embryo transfer is known.

### Ultrasound assessment

Ultrasound scans were performed using GE Voluson™ E6 or E8 ultrasound machines (GE Healthcare, Zipf, Austria), equipped with a RAB4‐8‐D 3D/4D curved‐array abdominal transducer (4–8.5 MHz), and were conducted by FMF fetal medicine fellows trained in the technique of 3D‐FTPV acquisition. Ultrasound machines were configured in advance with the correct settings, and these were saved as a preset mode to ensure consistency (the settings used can be found in Table S1). Viability of the pregnancy was confirmed, and CRL and nuchal translucency measurements were obtained, according to routine protocols. Subsequently, the placental ultrasound scan was performed by first identifying the ideal probe placement for 3D imaging of the entire placenta, which was typically a cross‐sectional plane near the placenta's center. Grayscale gain settings were adjusted to ensure that the placenta could be distinguished from the surroundings on visual assessment. A static grayscale volume was captured using preset machine settings, and the volume was reviewed to confirm that it included the whole placenta. If any part was missing or in a large shadow, the angle was adjusted or the probe was repositioned, and the capture was repeated to obtain a complete placental volume.

### Image processing

Images were downloaded directly from the ultrasound machines as .vol files without wavelet compression and were analyzed retrospectively by the Placental Imaging Research Group, Nuffield Department of Women's and Reproductive Health, University of Oxford, Oxford, UK. The placenta was segmented and the placental volume was calculated using the OxNNet toolkit, as described previously^11^, ^12^. In summary, OxNNet uses a fully convolutional neural network, trained on a ground truth of > 1000 segmented 3D ultrasound placental scans, to achieve fully automated placental segmentation. The placental images were subjected to a three‐stage quality control (QC) process, as detailed below.

#### Quality control Stage 1

Before placental segmentation, all images were reviewed retrospectively by trained research sonographers and assessed for completeness of placental capture. Images were excluded if the placenta was incomplete, the wrong machine settings were used, there was excessive power Doppler flash artifact (more than five large flashes) or the image quality was too poor to delineate the placenta by eye.

#### Quality control Stage 2

In the second stage of QC, placental and amniotic fluid segmentation were performed using both the current iteration of OxNNet and the original OxNNet prototype. The segmentation obtained by each method was compared using the Dice similarity coefficient. Cases with poor agreement were reviewed manually by two authors (S.M., M.So.), and those in which the placental contour could not be determined visually (e.g. overall very poor image quality) were excluded. Images were identified for review in three rounds: (1) agreement of placental segmentation < 60%, irrespective of agreement of amniotic fluid segmentation; (2) agreement of placental segmentation < 75% and agreement of amniotic fluid segmentation < 40%; and (3) agreement of amniotic fluid segmentation < 40%, irrespective of agreement of placental segmentation.

#### Quality control Stage 3

In the third stage of QC, outliers were identified based on the interquartile range (IQR) method for each day of gestation. For day of gestation, the data were subset and the first (Q1) and third (Q3) quartiles of placental volume measurement were calculated. Observations with volume measurements falling below Q1 − 1.5 × IQR or above Q3 + 1.5 × IQR were considered outliers. All identified outliers were reviewed manually by S.M. and M.So. to assess image quality and segmentation accuracy. Outliers were excluded if the image quality was insufficient to reliably determine the correct placental contour.

### Statistical analysis

Statistical analysis was performed using R version 4.3.1 and RStudio version 2024.09.0+375 (R Foundation for Statistical Computing Platform, Vienna, Austria).

#### Sample size estimation

The sample size of the FirstPLUS study was predetermined to ensure sufficient data for the construction of multivariable predictive models, allowing for attrition and loss to follow‐up. The minimum number of cases was calculated according to the method of Riley *et al*.^19^, using the *pmsampsize* version 1.1.3 package in R. Assuming a rare outcome (2% incidence), a model with 20 parameters and a C‐statistic of 0.9, 2171 cases would be sufficient to develop a multivariable predictive model. For a C‐statistic of 0.8, 2992 cases would be required. A recruitment target of 4000 cases was therefore selected pragmatically.

To determine whether the available cases (after exclusions) were sufficient for the construction of reference charts, we performed a calculation to assess the precision of the data in estimating specific centiles (2.5^th^ and 97.5^th^), using the following formula^20^:

$${\mathrm{SE}}_{p}=\mathrm{SD}\sqrt{\left(1+\frac{1}{2}{Z_{p}}^{2}\right)/n}$$

In this formula, SE_*p*_ is the standard error of the *p* ^th^ centile, SD is the standard deviation, *Z*_*p*_ is the *Z*‐score corresponding to the *p* ^th^ centile and *n* is the sample size. For a target SE of 0.05 at the 2.5^th^ and 97.5^th^ centiles (with a calculated SD of 1 after standardized Box–Cox transformation to ensure Gaussian distribution), a sample size of 1169 was estimated to be sufficient. Calculations based on the actual sample size after exclusions (*n* = 2547) and the calculated SD returned a SE of 0.034, indicating that the sample size is sufficient for centile chart construction. The choice of transformation method was determined using the *bestNormalize* package in R and, for the purposes of this calculation, we assumed no change in variance according to gestational age.

#### Models for reference chart construction

The distribution of FTPV was assessed visually using a histogram and a quantile–quantile (Q–Q) plot. Box‐and‐whiskers plots and faceted histograms were also constructed to assess changes in distribution with varying gestational age. As a result, we opted to explore flexible statistical methods capable of accommodating variation in distribution across gestational age, including quantile regression (QR)^21^ and the lambda‐mu‐sigma (LMS) method^22^, rather than the classical mean and SD approach^23^.

QR was performed using the *quantreg* package in R. The analysis involved fitting a variety of models, including linear models, polynomial models and models using natural splines and B‐splines. Both the Barrodale and Roberts (default linear programming) method and the penalized lasso regression method were applied to estimate the quantiles.

LMS modeling was performed using the *lms* function within the *gamlss* package in R, with models fitted using the default Rigby Stasinopoulos algorithm. A range of Generalized Additive Models for Location, Scale and Shape (GAMLSS) distribution families were assessed to identify the best‐fitting distribution for the data. The distribution families evaluated included the Box–Cox Cole and Green original (BCCGo), Box–Cox Power Exponential (BCPEo), Box–Cox *t*‐Distribution Extended (BCTEo), Box–Cox *t*‐Distribution (BCT), Skew Exponential Power (SEP1, SEP2, SEP3, SEP4), Sinh–Arcsinh (SHASH, SHASHo), Johnson's SU (JSU), Generalized *t*‐Distribution (GT), Log Normal (LOGNO) and Skew *t*‐Distribution Types 1 through 5 (ST1, ST2, ST3, ST4, ST5). The number of cycles was set to 1000, with a *k* value of 2 and all other parameters set to default.

Models for FTPV were constructed using both CRL (mm) and gestational age (days) as predictors.

#### Evaluation of models

More complex models may achieve better fit but at the cost of generalizability (overfitting). The Akaike information criterion (AIC) was selected as the method of model comparison because it provides a means of evaluating the goodness of fit, while penalizing against complexity^24^. Lower AIC values indicate a better balance between fit and parsimony. Charts were constructed with centile curves and raw data plots to confirm the goodness of fit of all potential models. Histograms and Q–Q plots of *Z*‐scores were also constructed.

#### Analysis of excluded cases

Comparisons were conducted between the main study cohort and the cases excluded during the third stage of QC. The baseline characteristics of each group were compared, and *P*‐values were calculated using the two‐sided Wilcoxon rank‐sum test for continuous variables and Fisher's exact test for discrete variables.

## RESULTS

**Figure 1:**
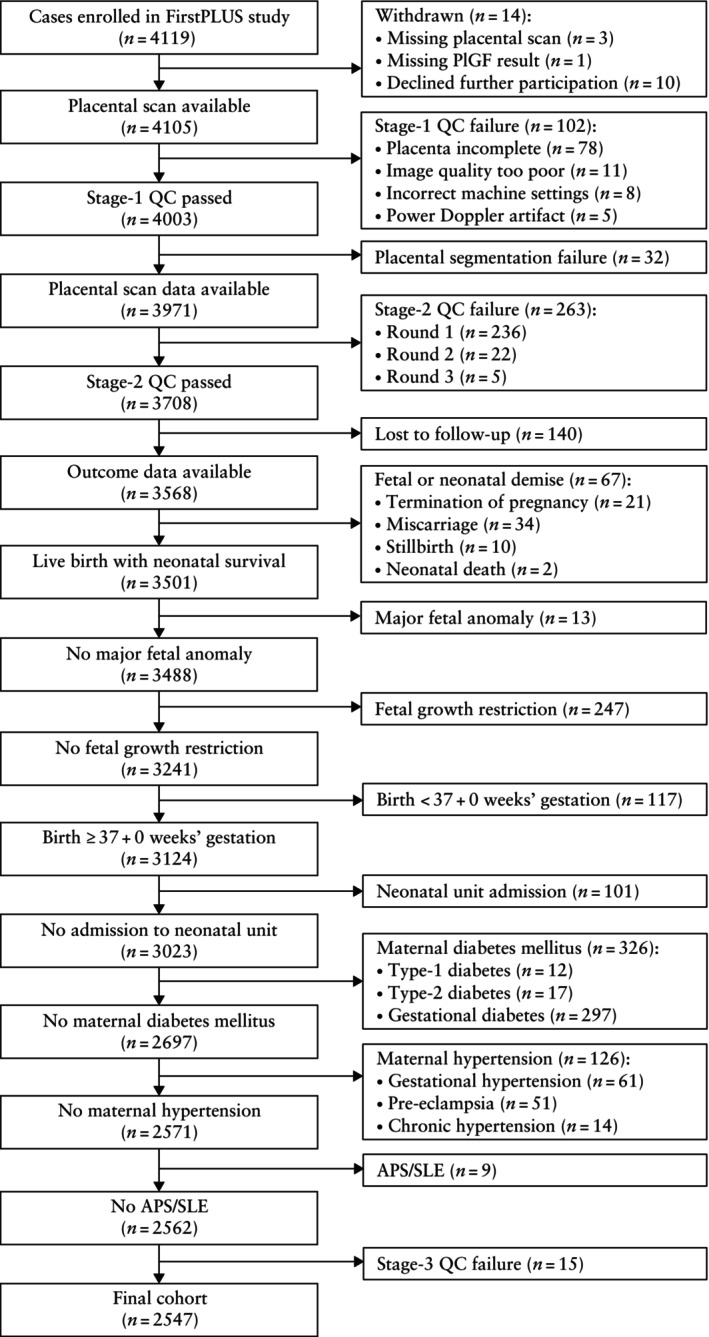
Flowchart summarizing selection of study cohort. APS, antiphospholipid syndrome; FirstPLUS, First Trimester Placental Ultrasound; PlGF, placental growth factor; QC, quality control; SLE, systemic lupus erythematosus.

After exclusions, the final cohort included 2547 cases. A flowchart demonstrating how this cohort was derived is provided (Figure 1) and the characteristics of the cohort are supplied (Table 1).

**Table 1:** Characteristics of 2547 singleton pregnancies included in study cohort

Variable	Value
Age (years)	33.4 (30.4–36.2)
Weight (kg)	66.6 (59.9–75.6)
Height (cm)	166.0 (162.0–170.8)
Body mass index (kg/m^2^)	23.9 (21.5–27.2)
Ethnicity	
Black	286 (11.23)
East Asian	52 (2.04)
South Asian	164 (6.44)
White	1944 (76.33)
Mixed	101 (3.97)
Mode of conception	
Spontaneous	2368 (92.97)
Ovulation drugs	14 (0.55)
*In‐vitro* fertilization	165 (6.48)
Smoker at presentation	46 (1.81)
Nulliparous	1209 (47.47)
Parous, previous PE	40 (1.57)
Parous, no previous PE	1298 (50.96)
Parous, previous FGR	181 (7.11)
Parous, no previous FGR	1157 (45.43)
Female fetal sex	1264 (49.63)
Gestational age at birth (weeks)	39.9 (39.0–40.7)
Birth weight (g)	3440 (3195–3740)
Birth‐weight centile	51.5 (30.2–74.7)
Birth weight < 10^th^ centile	122 (4.79)

Data are given as median (interquartile range) or *n* (%). FGR, fetal growth restriction; PE, pre‐eclampsia.

### Placental volume distribution

**Figure 2:**
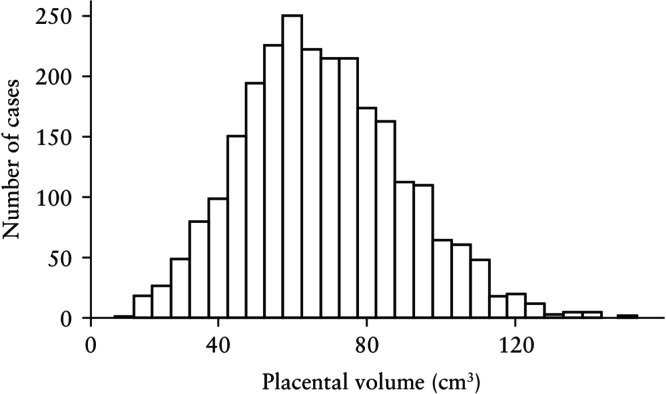
Histogram of first‐trimester placental volume in study cohort.

**Figure 3:**
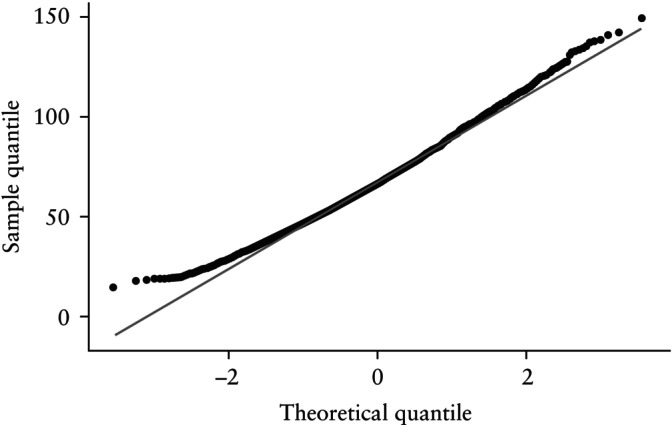
Quantile–quantile plot of first‐trimester placental volume in study cohort.

**Figure 4:**
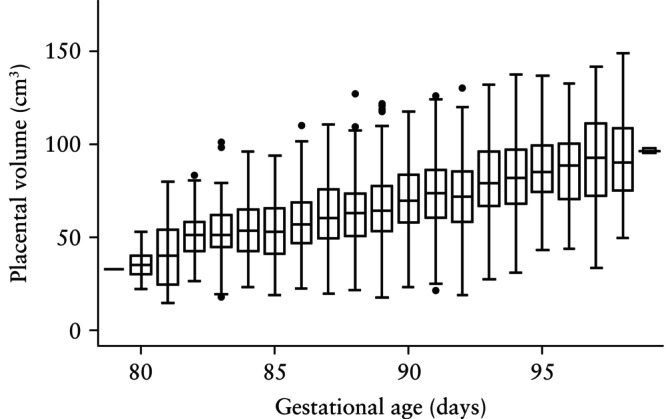
Box‐and‐whiskers plot of first‐trimester placental volume by gestational age. Boxes with internal lines are median and interquartile range (IQR), whiskers are 1.5 × IQR and circles are outliers.

Visual assessment of the histogram (Figure 2) and Q–Q plot (Figure 3) for FTPV across all gestational ages revealed a positively skewed distribution. Furthermore, visual assessment of a box‐and‐whiskers plot (Figure 4) and faceted histograms (Figure S1) by gestational age indicated that there was variation in the distribution of FTPV at different timepoints within the study window, although the interpretation of these results was limited at the extremes of gestational age by low case numbers.

### Comparison of models

Table 2 summarizes the AIC statistics for the QR models. When predicting FTPV from the CRL, the QR model with the lowest AIC value (22 429.98) employed a fourth‐order polynomial approach combined with the Barrodale and Roberts method. In contrast, the LMS method, using the SHASH distribution, achieved a substantially lower AIC of 22 134.33.

**Table 2:** Akaike information criterion (AIC) assessment of quantile regression models for prediction of first‐trimester placental volume from crown–rump length (CRL) or gestational age (GA)

	AIC	
Modeling approach/ methodology	CRL (in mm)	GA (in days)
Linear		
B&R	22 430.16	22 434.66
PLR	22 989.32	23 121.66
Polynomial 2		
B&R	22 431.11	22 434.43
PLR	22 517.84	22 511.89
Polynomial 3		
B&R	22 432.65	22 436.24
PLR	22 627.73	22 609.47
Polynomial 4		
B&R	22 429.98	22 434.15
PLR	22 856.40	22 744.46
Natural splines		
B&R	22 433.67	22 437.04
PLR	22 456.14	22 458.36
B‐splines		
B&R	22 431.16	22 434.78
PLR	22 506.26	22 481.40

B&R, Barrodale and Roberts method; PLR, penalized lasso regression.

For predicting FTPV from gestational age, the QR model with the lowest AIC value (22 434.15) used a fourth‐order polynomial approach combined with the Barrodale and Roberts method. However, the LMS method, based on the BCCGo distribution, produced the lowest AIC of 22 135.04.

### Final models

**Figure 5:**
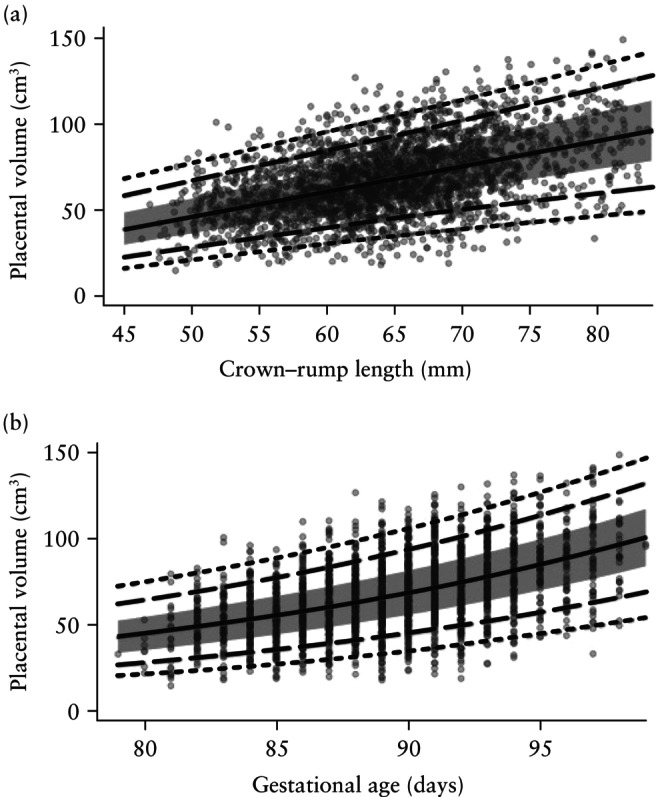
Reference chart for first‐trimester placental volume modeled against (a) crown–rump length and (b) gestational age. Solid line, 50^th^ centile; grey band, 25^th^–75^th^ centile; dashed lines, 10^th^ and 90^th^ centiles; dotted lines, 3^rd^ and 97^th^ centiles.

Owing to their lower AIC values, the models generated by the LMS method were selected to construct the centile charts. The resulting centile charts for FTPV according to CRL and gestational age are presented in Figure 5a and 5b, respectively.

The model summaries, including coefficients, are provided in Appendix S1. The two models are also available as an R file (Appendix S2), and the R script for the calculation of individual centiles is also provided (Appendix S3).

### Evaluation of centile charts

A comparison of the predicted and observed centiles for the two models across a range of target centiles (1^st^, 3^rd^, 10^th^, 25^th^, 50^th^, 75^th^, 90^th^, 97^th^ and 99^th^ centiles) is provided in Table 3. Both models demonstrated strong agreement between the centiles predicted from calibration and the observed centiles in the sample, with minor deviations. Performance was similar for both models with respect to the comparison of observed centiles with predicted centiles, with deviations of ≤ 0.5 percentage points across all target centiles evaluated. Similar patterns of close agreement were observed at the extremes.

**Table 3:** Percentage of cases at various centiles of first‐trimester placental volume observed in study cohort *vs* predicted by final lambda‐mu‐sigma models based on crown–rump length (CRL) or gestational age (GA)

		Predicted from calibration (%)	
Target centile	Observed in sample (%)	CRL model	GA model
1^st^	1.02	0.866	0.897
3^rd^	3.02	2.673	2.602
10^th^	10.01	9.706	9.908
25^th^	25.01	24.856	25.102
50^th^	50.02	49.608	49.612
75^th^	74.99	75.284	75.332
90^th^	89.99	89.743	90.010
97^th^	96.98	96.857	96.933
99^th^	98.98	99.041	98.934

**Figure 6:**
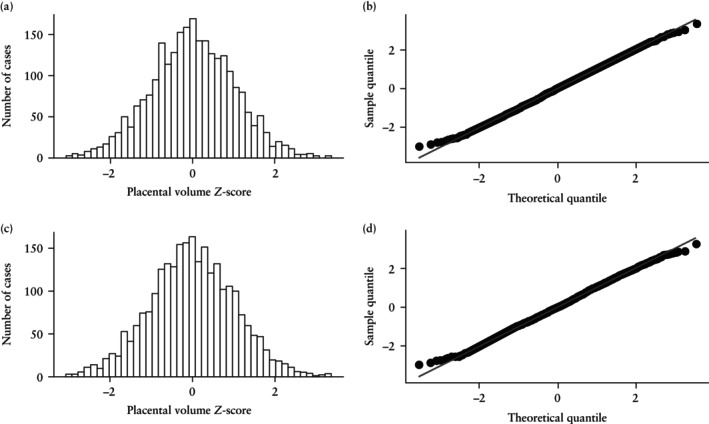
Histograms (a,c) and quantile–quantile plots (b,d) showing distribution of *Z*‐scores for first‐trimester placental volume according to crown–rump‐length model (a,b) and gestational‐age model (c,d).

The *Z*‐scores calculated with each of the final models are presented as histograms and Q–Q plots (Figure 6), demonstrating the expected normal distribution in both cases.

### Analysis of excluded cases

Comparison of demographic characteristics between the study cohort and the 15 cases excluded in the third stage of QC is supplied in Table S2. In all comparisons but one, no statistically significant difference was observed between groups. The exception was a history of pre‐eclampsia, which was more common among the excluded cases (2/15 (13.3%)) compared with the study cohort (40/2547 (1.6%)) (*P* < 0.001).

## DISCUSSION

This study presents reference ranges for FTPV, modeled against CRL and gestational age, based on a large cohort of healthy pregnancies. We use advanced machine learning, specifically the OxNNet toolkit, for fully automated placental segmentation and volume calculation. We recommend the CRL‐based model where possible, as it provides more precise modeling compared with the gestational‐age‐based model, which groups placental volume by gestational age in days and may introduce artificial clustering. However, both models showed equivalent performance when comparing observed and predicted FTPV centiles. The gestational‐age‐based model offers flexibility for cases in which placental volume is measured later in gestation, making it useful for both clinical and research applications. The higher prevalence of pre‐eclampsia in the cases excluded during the third stage of QC is likely a statistical artifact due to the small sample size (*n* = 15). This is unlikely to impact the construction of placental volume centile charts, as the overall cohort remains representative.

Our study is not the first to produce reference ranges for FTPV. Previous studies, such as those of Meengeonthong *et al*.^25^, Trilla Solà *et al*.^26^ and Guyomard *et al*.^27^, have attempted to establish similar reference ranges. Meengeonthong *et al*. constructed FTPV reference charts using a cohort of 236 low‐risk pregnancies, relying on virtual organ computer‐aided analysis (VOCAL™) software (GE Healthcare) for placental segmentation and volume calculation^25^. Their method employed the mean and SD approach for reference range construction, modeling FTPV against CRL. Although the coefficients are provided, the cohort characteristics were not described clearly, and it is therefore difficult to assess the applicability of their reference ranges to a wider population. Guyomard *et al*. performed a feasibility study involving 128 cases, also using VOCAL for volume calculation^27^. The mean and SD‐based approach was used to model FTPV against CRL, but the SD was treated as a constant, which may limit model accuracy because of the changing distribution shape with gestational age (a finding corroborated by all three of the studies mentioned here). Trilla Solà *et al*. published charts of placental volumes corresponding to the 5^th^, 10^th^, 50^th^, 90^th^ and 95^th^ centiles according to both CRL and GA^26^. These were derived from a cohort of 1142 uncomplicated pregnancies, and segmentation was performed manually, which has so far been the reference standard. However, the coefficients needed for calculating *Z*‐scores or centiles from raw measurements were not provided, limiting clinical utility.

In comparison, our study benefits from a much larger sample size, more than double that of the largest previous study^26^, enabling greater precision in estimating FTPV distributions across gestational ages. This enhances the reliability of centile calculations, particularly for extreme centiles, such as the 5^th^ and 95^th^ centiles. The larger sample also ensures better representation of subgroups, improving the generalizability of the charts. With more data points, the influence of outliers is minimized, resulting in more robust centile charts that better reflect the true population distribution.

In contrast to previously published reference ranges, we used the more advanced LMS method to model FTPV, accounting for its complex, non‐normal distribution, which varies with gestational age. This approach offers greater flexibility compared with the simpler mean and SD method. Although the LMS method carries a risk of overfitting, we mitigated this by visually inspecting the curves and using the AIC to assess model fit, ensuring robustness without excessive complexity.

A key strength of our study is the use of the OxNNet toolkit. The availability of fully automated placental segmentation and volume calculation has facilitated the creation of a larger dataset than has previously been feasible using the reference standard of manual placental segmentation. The OxNNet toolkit also offers a significant advantage over the VOCAL method, which relies on geometric assumptions that may not accurately represent the irregular shape of the placenta, especially in the first trimester^28^. Work has shown that the shape of the placenta in early pregnancy can vary considerably^29^, potentially making manual and geometric methods of segmentation prone to errors in volume estimation.

Another strength is the rigorous QC applied to both placental imaging and segmentation, ensuring that our models are based on the most accurate placental volume assessments. For FTPV to be a reliable clinical marker, maintaining high image quality is crucial. To support this, we are developing integrated QC features within the OxNNet toolkit, enabling automated, real‐time QC during image capture. This will allow sonographers to receive immediate feedback and to repeat scans if issues such as incomplete images or significant artifacts arise, thereby enhancing the reliability of FTPV measurements in clinical practice.

However, there are some limitations to consider. Although our cohort is large, there is a relative paucity of cases at the extremes of gestational age, making the distributions harder to interpret at these timepoints. Including more data from earlier and later gestational ages within the first trimester would improve the robustness of the models. Additionally, our study is based on a specific population attending first‐trimester combined screening at a single UK center, which limits the generalizability of our findings to other populations with different demographic profiles. Further studies conducted in more diverse cohorts are needed. Although we excluded cases with known fetal or maternal pathology, undiagnosed or subclinical conditions may still have influenced placental volume, highlighting the need for further research to explore the relationship between FTPV and pregnancy outcome. Placental location and uterine fibroids do not affect segmentation quality, but this would benefit from further investigation. The performance of the OxNNet toolkit with different ultrasound machines should also be evaluated. Another limitation is the complexity of the LMS approach compared with the traditional mean and SD method. Although the LMS method offers more accurate modeling of placental volume distribution, its application requires a higher level of mathematical expertise. To facilitate wider adoption, we have made the models and R scripts for centile calculation available in the supplementary material, ensuring that other researchers can replicate and apply our findings.

In conclusion, this study defines reference ranges for FTPV, based on CRL and gestational age, in a large cohort of healthy pregnancies. Using artificial intelligence software for automated placental segmentation, we established robust, reproducible FTPV reference ranges for clinical and research use. The CRL‐based model is preferred, though the gestational‐age‐based model may be useful when the CRL is unavailable. This work lays the foundation for future research on FTPV and pregnancy outcome.

## Supporting information


**Table S1** Ultrasound machine settings used in study

**Table S2** Comparison of demographic characteristics between study cohort and 15 cases excluded in third stage of quality control

**Figure S1** Faceted histograms for first‐trimester placental volume for each day of gestation.

**Appendix S1** Model summary for prediction of first‐trimester placental volume based on crown–rump length or gestational age

**Appendix S2** RData file containing models for centile calculation based on crown–rump length or gestational age

**Appendix S3** R file including relevant scripts to perform centile calculations

## Data Availability

The data that support the findings of this study are available on request from the corresponding author. The data are not publicly available due to privacy or ethical restrictions.
