# Supplementary material for: Reference charts for first‐trimester placental volume derived using OxNNet
Source: Ultrasound Obstet Gynecol. 2025 Aug 1;66(3):337–46. doi: 10.1002/uog.29300 (PMC12401500; doi:10.1002/uog.29300)
Supplement: Supplementary file 1 — Table S1 Ultrasound machine settings used in study [file UOG-66-337-s004.docx]

**Table S1:** Ultrasound machine settings

| **Menu** | **Parameter** | **Setting** |
| --- | --- | --- |
| Main 2D | XBeam Compound receive imaging | Off |
|  | Speckle reduction imaging | 3 |
|  | Gray map | 16 |
|  | Tint Map | Off |
|  | Angle | 90° |
|  | Time gain compensation | Far Field |
| Sub 2D | Gray map | 16 |
|  | Tint | Off |
|  | Line filter | High |
|  | Persistence | 6 |
|  | Enhance | 2 |
|  | Line density | Normal |
|  | Reject | 20 |
|  | Output thermal index | Normal |
| 3D | Volume angle | 85° |
|  | Quality | High1 |
