# Supplementary material for: Reference charts for first‐trimester placental volume derived using OxNNet
Source: Ultrasound Obstet Gynecol. 2025 Aug 1;66(3):337–46. doi: 10.1002/uog.29300 (PMC12401500; doi:10.1002/uog.29300)
Supplement: Supplementary file 2 — Table S2 Comparison of demographic characteristics between study cohort and 15 cases excluded in third stage of quality control [file UOG-66-337-s005.docx]

**Table S2:** Comparison of demographic characteristics between study cohort and 15 cases excluded in third stage of quality control

| **Variable** | **Study cohort**  **N = 2547**  N (%) or Median (IQR) | **Excluded outliers**  **N = 15**  N (%) or Median (IQR) | **P value** |
| --- | --- | --- | --- |
| Maternal age (years) | 33.4 (30.4–36.2) | 32.0 (27.9–33.2) | 0.06 |
| Maternal weight (kg) | 66.6 (59.9–75.6) | 70 (55.0–78.2) | 0.94 |
| Maternal height (cm) | 166 (162–171) | 166 (160–173) | 0.89 |
| Body mass index | 23.9 (21.5–27.2) | 25.4 (21.0–26.6) | 0.96 |
| Ethnicity |  |  | 0.21 |
| Black | 286 (11.2) | 3 (20.0) |  |
| East Asian | 52 (2.0) | 1 (6.7) |  |
| South Asian | 164 (6.4) | 0 |  |
| White | 1944 (76.3) | 10 (66.7) |  |
| Mixed race | 101 (4.0) | 1 (6.7) |  |
| Conception |  |  | 0.65 |
| Spontaneous | 2368 (93.0) | 15 (100.0) |  |
| Ovulation drugs | 14 (0.6) | 0 |  |
| In vitro fertilization | 165 (6.5) | 0 |  |
| Parity |  |  | 0.44 |
| Nulliparous | 1209 (47.5) | 9 (60.0) |  |
| Multiparous | 1338 (52.5) | 6 (40.0) |  |
| Smoker at booking | 46 (1.8) | 0 | NA |
| History of previous pre-eclampsia |  |  | <0.001 |
| Previous pre-eclampsia | 40 (1.6) | 2 (13.3) |  |
| Multiparous, no pre-eclampsia | 1298 (51.0) | 4 (26.7) |  |
| Previous FGR (birthweight <3^rd^ centile) |  |  | 0.22 |
| Previous FGR | 181 (7.1) | 2 (13.3) |  |
| Multiparous, no FGR | 1157 (45.4) | 4 (26.7) |  |
| Sex |  |  | 0.12 |
| Female | 1264 (49.6) | 4 (26.7) |  |
| Male | 1283 (50.4) | 11 (73.3) |  |
| Gestational age at birth (weeks) | 39.9 (39.0–40.7) | 39.7 (38.4–39.9) | 0.05 |
| Birthweight (g) | 3440 (3195–3740) | 3200 (3065–3685) | 0.21 |
| Birthweight (centile) | 51.5 (30.2–74.7) | 53.6 (32.0–71.3) | 0.98 |
| Birthweight <10^th^ centile | 122 (4.8) | 1 (6.7) | 0.52 |
