# Supplementary figures and images for: Reference charts for first‐trimester placental volume derived using OxNNet
Source: Ultrasound Obstet Gynecol. 2025 Aug 1;66(3):337–46. doi: 10.1002/uog.29300 (PMC12401500; doi:10.1002/uog.29300)

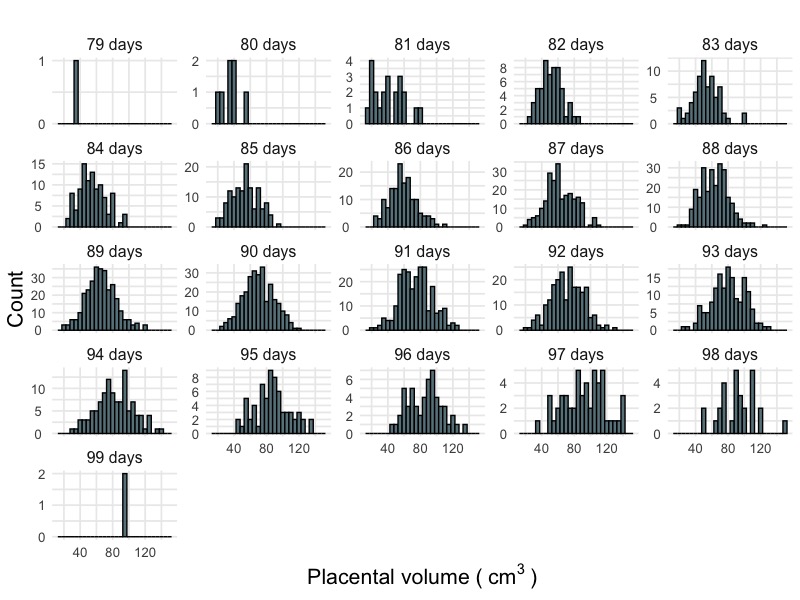

Supplement: Supplementary file 3 — Figure S1 Faceted histograms for first‐trimester placental volume for each day of gestation. [file UOG-66-337-s002.jpeg]
