# Supplementary material for: Reference charts for first‐trimester placental volume derived using OxNNet
Source: Ultrasound Obstet Gynecol. 2025 Aug 1;66(3):337–46. doi: 10.1002/uog.29300 (PMC12401500; doi:10.1002/uog.29300)
Supplement: Supplementary file 4 — Appendix S1 Model summary for prediction of first‐trimester placental volume based on crown–rump length or gestational age [file UOG-66-337-s001.docx]

**Appendix S1**

Model summary for prediction of placental volume (mm^3^) from crown-rump length (CRL) (mm)

******************************************************************

Family: c("SHASH", "Sinh-Arcsinh")

Call:

gamlss(formula = vol ~ pb(CRL), sigma.formula = ~pb(CRL), nu.formula = ~pb(CRL),

, tau.formula = ~pb(CRL), familvol = "SHASH", data = dataTEMP,

, n.cvolc = 1000)

Fitting method: RS()

------------------------------------------------------------------

Mu link function: identity

Mu Coefficients:

Estimate Std. Error t value Pr(>|t|)

(Intercept) -28.0265 3.2845 -8.533 <2e-16 ***

pb(x, df = mu.df) 1.4791 0.0518 28.557 <2e-16 ***

---

Signif. codes: 0 ‘***’ 0.001 ‘**’ 0.01 ‘*’ 0.05 ‘.’ 0.1 ‘ ’ 1

------------------------------------------------------------------

Sigma link function: log

Sigma Coefficients:

Estimate Std. Error t value Pr(>|t|)

(Intercept) 1.784989 0.131770 13.546 <2e-16 ***

pb(x, df = sigma.df) 0.019137 0.002025 9.449 <2e-16 ***

---

Signif. codes: 0 ‘***’ 0.001 ‘**’ 0.01 ‘*’ 0.05 ‘.’ 0.1 ‘ ’ 1

------------------------------------------------------------------

Nu link function: log

Nu Coefficients:

Estimate Std. Error t value Pr(>|t|)

(Intercept) 0.396027 0.160329 2.470 0.0136 *

pb(x, df = nu.df) -0.005305 0.002464 -2.153 0.0314 *

---

Signif. codes: 0 ‘***’ 0.001 ‘**’ 0.01 ‘*’ 0.05 ‘.’ 0.1 ‘ ’ 1

------------------------------------------------------------------

Tau link function: log

Tau Coefficients:

Estimate Std. Error t value Pr(>|t|)

(Intercept) -0.749386 0.135089 -5.547 3.20e-08 ***

pb(x, df = tau.df) 0.011469 0.002092 5.482 4.62e-08 ***

---

Signif. codes: 0 ‘***’ 0.001 ‘**’ 0.01 ‘*’ 0.05 ‘.’ 0.1 ‘ ’ 1

------------------------------------------------------------------

NOTE: Additive smoothing terms exist in the formulas:

i) Std. Error for smoothers are for the linear effect only.

ii) Std. Error for the linear terms may not be reliable.

------------------------------------------------------------------

No. of observations in the fit: 2547

Degrees of Freedom for the fit: 8.011393

Residual Deg. of Freedom: 2538.989

at cycle: 101

Global Deviance: 22118.3

AIC: 22134.33

SBC: 22181.14

******************************************************************

Model summary for prediction of placental volume (mm^3^) from gestational age (GA) (days)

******************************************************************

Family: c("BCCGo", "Box-Cox-Cole-Green-orig.")

Call:

gamlss(formula = vol ~ pb(GA), sigma.formula = ~pb(GA), nu.formula = ~pb(GA),

, tau.formula = ~pb(GA), familvol = "BCCGo", data = dataTEMP,

, n.cvolc = 1000)

Fitting method: RS()

------------------------------------------------------------------

Mu link function: log

Mu Coefficients:

Estimate Std. Error t value Pr(>|t|)

(Intercept) 0.363378 0.130506 2.784 0.0054 **

pb(x, df = mu.df) 0.042953 0.001453 29.560 <2e-16 ***

---

Signif. codes: 0 ‘***’ 0.001 ‘**’ 0.01 ‘*’ 0.05 ‘.’ 0.1 ‘ ’ 1

------------------------------------------------------------------

Sigma link function: log

Sigma Coefficients:

Estimate Std. Error t value Pr(>|t|)

(Intercept) 0.0004063 0.3440516 0.001 0.999058

pb(x, df = sigma.df) -0.0142152 0.0038484 -3.694 0.000226 ***

---

Signif. codes: 0 ‘***’ 0.001 ‘**’ 0.01 ‘*’ 0.05 ‘.’ 0.1 ‘ ’ 1

------------------------------------------------------------------

Nu link function: identity

Nu Coefficients:

Estimate Std. Error t value Pr(>|t|)

(Intercept) -1.25063 1.30178 -0.961 0.337

pb(x, df = nu.df) 0.02300 0.01462 1.573 0.116

------------------------------------------------------------------

NOTE: Additive smoothing terms exist in the formulas:

i) Std. Error for smoothers are for the linear effect only.

ii) Std. Error for the linear terms may not be reliable.

------------------------------------------------------------------

No. of observations in the fit: 2547

Degrees of Freedom for the fit: 6.031686

Residual Deg. of Freedom: 2540.968

at cycle: 5

Global Deviance: 22122.97

AIC: 22135.04

SBC: 22170.28

******************************************************************
